# Collective eating and the management of chronic disease in Dakar: translating and enacting dietary advice

**DOI:** 10.1080/09581596.2021.1898545

**Published:** 2021-03-11

**Authors:** Branwyn Poleykett

**Affiliations:** Sociology, Philosophy and Anthropology, University of Exeter, Exeter, UK

**Keywords:** Consumption, health behaviour, older people

## Abstract

In the past decade, Non-Communicable Diseases (NCDs) have become a highly visible public health issue in Senegal. In the absence of adequate and affordable care, people diagnosed with NCDs seek to manage their symptoms through the adoption of healthy diet. However, in households built on collective eating, dietary change is extremely challenging. Drawing on participant observation, biographical interviews, and focus groups with women in six households in the Dakar suburb of Pikine, this paper presents a relational analysis of the reception and translation of dietary advice within low-income households. Women diagnosed with chronic disease strategically ‘bracketed’ advice that was not possible to enact, prioritised collective transformation over individual change, and valued consumption that demonstrated ‘respect’ and solidarity over ‘healthy eating’. I show that relational approaches open up new intervention and health promotion strategies for the prevention and management of Non-Communicable Diseases outside of the global North.

## Introduction

Non-Communicable Diseases, in particular hypertension and diabetes, are increasingly common among people in middle and older age in Senegal (Macia et al., 2016; Ndiaye et al., 2016; Vialard et al., 2017; Walker et al., 2019). In the low-income, food-insecure households in which I conducted my research, diagnoses of hypertension and diabetes were almost ubiquitous and the majority of older people I spoke to told me that they were taking steps to eat a healthy diet and improve their health. However, making these changes was, in practice, extremely challenging. As in many parts of the world, an ‘ideal’ healthy diet was unaffordable for poorer Senegalese (Hirvonen et al., 2020). The task of nourishing an older person diagnosed with a chronic disease was challenging and, as older people were acutely aware, diabetes and hypertension were not the only manifestation of diet-related debility and disruption present in urban families. The households that I discuss in this paper were affected by multiple forms of malnourishment (Branca et al., 2019); under-nutrition, micronutrient deficiencies and anaemia were all present, affecting the health of household members at all stages of the lifecourse, and the younger women responsible for cooking shared meals and nourishing the family were confronted with the significant task of producing an adequate amount of food that met the needs of everyone in the household. This was further complicated by the fact that in Dakar, as Emily Yates-Doerr writes of households in Guatemala, women ‘did not cook for bodies, but for diffuse and shifting collectives’ (Yates-Doerr, 2017, p. 143). In a context where all food is shared and the act of collective eating carries a strong moral weight and cultural significance, there is significant pressure for everyone to eat from the collective bowl and crafting an individual ‘diet’ is an extremely complex undertaking. People who attempt to break off from collective eating to adhere to the diet they have been advised to eat risk their reputation and social status as well as their relationships with their family (Rubin & BeLue, 2017).

In this paper, I use a relational approach to examine how chronic disease impacts on and reshapes intra-household bargaining, the process of negotiating equitable food allocation among household members. Attempts by individuals to enact dietary advice can result in changes to the organisation of the collectivities in which they are embedded. Research participants who sought to change the way that food was sourced, prepared and shared found that they often triggered intense conflict, not only over the material question of what to eat, but over social questions of which family member holds the authority over food preparation and consumption. In demonstrating how individual consumption is inextricably entangled in a complex and often conflicted context of communal eating, I build upon and extend the critique of individualising and behaviourist public health perspectives embedded in the Social Determinants of Health Approach. The SDoH approach provides an acute analysis of how Non-Communicable Diseases emerge and shape opportunities for health, analysing the relationship between structural and individual level drivers of ill health. This approach decenters individual responsibility, producing a complex picture of how agency and health behaviours are shaped by ‘upstream’ factors such as the social and economic context within which people live, the built environment they negotiate, and the healthcare systems to which they have access. Focusing on individual behaviour does not just shift responsibility for maintaining health onto individuals, it unhelpfully narrows disease etiologies, obscuring how day-to-day choices are constrained by larger structural factors (Gálvez, 2020). Despite the significant impact of SDoH approaches in transforming public health approaches, researchers have pointed to a ‘lifestyle drift’, the process through which upstream factors are translated and operationalised into behaviour change programmes targeting individuals (Glasgow & Schrecker, 2015). One good example of this ‘drift’ is the EAT/Lancet Planetary Diet project, which appraises long term and structural changes in food systems and dietary patterns and translates that data in a package of dietary advice to be enacted by individuals. Moreover, the SDoH approach has paid relatively less attention to the familial, communal, public, and collective structures that mediate between individuals and society (Russell et al., 2018). Research in sub-Saharan Africa had shown that family support plays a key role in supporting behaviour change (BeLue, 2016).

This paper focuses closely on minute and mundane household interactions, building on the idea of dietary change, chronic management, and self-care as relational ‘homework’, a form of practice defined as ‘an extensive social project that generates new and unanticipated demands and relationships among family members’ (Mattingly et al., 2011). Women’s attempts to ‘control’ symptoms or to make changes to what they eat are assembled in relation to the need of others to eat suitable, safe, and sustaining food. Relational approaches open up spaces of community engagement over dietary transformation that can offer new pathways for public health responses to the management and control of diet-related chronic disease. I show how a fuller experiential and consensual understanding of dietary choice could be orchestrated between a range of possible intersectoral public health interventions to support dietary change and social transformation.

## Theoretical approach: relational theory

The ideal and imagined object of a dietary intervention is often taken to be an individual, socialised into an understanding that they possess a ‘diet’, exercising agency and choice, and identified with a ‘sovereign body’ (Guthman et al., 2014). Annemarie Mol in her account of diabetes care in the Netherlands shows that the idea of individual patients exercising choice and exerting responsibility over their state of health is a fantasy based in public health techniques of aggregation and optimisation rather than in reality. In fact, in clinical practice, a good deal of effort goes into ‘disentangling’ patients from their social environments (Mol, 2008, p. 58). Senegalese clinicians often participate in this process when they use visual aids such as nutrition wheels, or images of an ‘ideal plate’ to communicate with patients who eat collectively from a shared bowl and whose dietary ‘choices’ are inextricably linked with the preferences and priorities of other members of their households. Mol argues that people are embedded in collectives of different shapes and sizes and possibilities for health are shaped by the functioning of these collectives. What is important about these arguments for the purpose of analysing the logics that shape the distribution of food, care, and good health in Senegalese households is Mol’s suggestion that we do not make assumptions about how the character of the collectives relates to or produces certain kinds of care. Rather, we should empirically investigate how frictive interactions between individuals and collectives shape opportunities for health, while, at the same time, remaining attentive to how collectives have been historically formed. The long history of public health interventions to socially engineer diets in Senegal, the complex colonial histories of the West African ‘household’, the collective and eclectic cultural scripts that people mobilise to construct information about healthy diets, as well as the power inequalities between genders and generations that shape individual relationships with collectives, these are all elements that shape becoming and belonging within collectives in Dakar.

Relational sociology draws attention to the dynamic, processual, and unfolding nature of the social world. Applied to health and illness, the analytic advantage of a relational perspective is that it can show that agency is not a property of an individual ‘but instead inheres in relations between individuals in spatial contexts’ (Veenstra & Burnett, 2016). Moving beyond a broad interest in context sensitivity, relational approaches see individuals as reciprocally composed by relations with others, with institutional fields of knowledge and practice, and with environments and places. Although relational theory is associated with the sociology of kinship and intimate life, it does not imply a straightforward description of social relationships (Roseneil & Ketokivi, 2016). Rather, bringing relational theory to bear on data from urban households shows how a biomedical and ‘nutritionist’ appraisal of food fails to account for its relational significance: the way it composes, recomposes, materialises, dramatizes, and exposes the value that people place on and express through their relationships. Coupled with ethnographic data, relational analysis produces a fuller picture of the vagaries of ‘healthy eating’, for example, this paper shows that women often deviate from ‘healthy’ patterns of eating in order to realise their aspirations to invest in the care and nourishment of their kin, or to demonstrate their willingness to subordinate their nutritional needs to the desires of the collective. In other words, a relational approach has the analytic advantage of opening up experiential understandings of how people live with chronic conditions in a highly insecure context, as well as explaining strategies of ‘control’ and behaviours embedded in the social logics of West African households. A relational lens coupled with detailed ethnographic data can move beyond examining social relations as enabling or constraining of states of health, exposing instead the relational and intersubjective agency that is brought to bear on evaluating and enacting dietary change (Veenstra & Burnett, 2016). This data reinforces the finding that effective public health intervention and communication should be targeted at collectives rather than individuals, and I conclude the paper with a few suggestions about how to maximise the latitude of intersubjectively negotiated agency through materially supporting the spending power of the household.

## Materials and methods

This paper is based on four months of participant observation in households in Dakar, six focus groups and six follow-up in-depth biographical interviews. The participant observation was conducted in a large, multigenerational household in the suburb of Pikine and consisted of shopping, cooking, and eating with women. The selection of this method meant that men were excluded from this research as cooking and eating were gender-segregated activities and women were wholly responsible for converting the household’s fluctuating income into adequate and acceptable food. The first phase of participant observation opened up a rich understanding of the ethos of everyday eating and helped to provide a fuller and more realistic picture of realities of household economy and care that were often difficult to elicit using discursive methods. Detailed fieldnotes were taken and coded for emerging perspectives. When coding the different sources of data a general inductive approach was used to remain open to the perspectives and priorities of the research participants. In addition to in-depth data collection at one research site, a further six households were selected for further research. These households were roughly the same size (containing around 20–25 people) and each had a similar socio-economic status, although each household is unique and, as Senegalese households expand to incorporate people at different times, at various points in the research process household size and forms of family organisation changed. These fluctuations reflect the instability of life in a precarious milieu. The households were all in Pikine but they were chosen deliberately to represent a wide geographical spread and to show the heterogeneity of a ‘peri-urban’ area. Sometimes described as a suburb of Dakar, Pikine is home to over a million people and includes densely populated neighbourhoods closely linked to the economy of Dakar and more rural neighbourhoods. The more urban households, for example, were reliant on local markets for sourcing food, whereas the households in the more rural zones had some access to land.

A focus group was conducted with 6–8 women in each household. The criterion for inclusion in the focus group was that people were involved in the preparation of food for the household, which meant that participation was limited to women. The focus groups were conducted in Wolof and were recorded and analysed. The discussion in the focus groups examined people’s explanations for the emergence and origins of ‘new diseases’ [*feebaru yu bes*] (hypertension, heart disease, and diabetes). Participants were asked where they thought that ‘new diseases’ came from, who was most affected by them, and how they could be prevented or controlled. The choice of focus groups as a methodology reflected my evolving perception, drawing on preliminary participant observation, that the management of chronic disease was a social process, involving cooperation and consensus seeking between the generations and negotiation between people who prepared food and people who ate it. The focus group discussions generated particularly pertinent data because they shed light not only on social norms and normativity but also on how those norms are ‘negotiated, constructed, and legitimized’ (Kristiansen & Grønkiaer, 2018). For example, during the focus group discussions younger women resisted the implication from older women that the rise in chronic disease could be attributed to their ‘bad’ food preparation techniques and lack of respect and care for their kin, stressing that they had to balance the nourishment of the elderly with a range of other obligations, including the necessity of providing culturally significant rice-based dishes, the mainstay of the urban diet. While this dialogic approach yielded rich and complex data about how women related to one another, it should be considered a limitation of the current data that it does not take account of men’s understanding of their role in maintaining the health and wellbeing of aging relatives.

Relationships with the six households under study were maintained via informal visits and discussions over multiple fieldwork trips. After coding and analysing the focus group data I returned to each of the households to conduct in-depth, semi-structured, life history interviews with one woman in each household who was living with and managing the symptoms of chronic disease. These individuals were identified for follow-up research through both the focus groups and informal conversations and all of them agreed to talk about their lives and share their diagnoses. Three of the six interviews were with people who were in the original focus group and three were with women from the household who had not participated. These interviews were biographical and we encouraged research participants to reflect on their experiences of eating across their lifetimes. This research strategy emerged from the first round of analysis of the focus group data and ongoing participant observation. The purpose of this was partly to test an emerging theoretical proposition. Older women had, in the course of their lives, lived through significant food crises, and people often framed the ‘crisis’ of chronic disease as one in a series of ruptures in access to food and disruption to the established lexicon of eating. The life history interviews helped to illuminate how women’s understanding of eating and health had been shaped by biographical experiences like childbirth, breastfeeding, rural to urban migration and becoming part of different households, collectives, and communities with different eating norms and this helped to contextualise naturalised beliefs about food and eating within a longer timeframe.

## Results

The first theme that emerged from the focus group discussions was that people blamed high rates on chronic disease on what they called ‘bad eating’ [*lekkin bu bon*]. This broad consensus reflects an understanding that in most cases preceded diagnosis, that the standard, urban Senegalese diet was ‘bad’ and that Senegalese were generally stubborn in refusing to implement changes that could improve their health. Women observed extremely high rates of chronic disease in their households and communities, indeed, almost everyone I spoke with in middle and older age identified themselves as living with multiple chronic diseases. This situation was contrasted with the past when, as one focus group participant said, diabetes was known as *feebaru patron* or the boss’ disease, and associated exclusively with Dakar’s wealthy Lebanese population. When women received their diagnoses they were instructed to make changes to their diet and to increase their physical activity. For women in particular the option of increasing physical activity was extremely intimidating. Living in neighbourhoods that were polluted, informal, and perceived as insecure, most of the older women involved in the research dismissed this guidance as unrealistic. In contrast, women identified the dietary advice they were given as highly credible. In many cases, they believed that if they were able to change their diet and conform to the guidelines they had been given they would be ‘cured’. Even where women struggled to comply with the diet, in focus groups and in interviews women agreed that the diet that was proscribed was ‘healthy’, and ‘ideal’ and in cases where dietary change yielded imperfect or partial results, women were more likely to blame their own patchy compliance than to question the appropriateness of the dietary advice.

In identifying food practices that were harmful, women repeated the advice that they had been given that identified the three major culprits: fat, salt, and sugar. Fat, particularly in the form of *diwlin* or cheap vegetable oil was identified as particularly suspicious, partly because women associated it with substitution campaigns in the past. Other food items were identified as harmful because of their sensory properties and high flavour, like chillies. The dietary advice they were given was interpreted as a rupture with familiar and pleasurable eating habits and the imposition of sometimes drastic restrictions. The majority of research participants reported that ‘healthy eating’ entailed a significant sacrifice As Vialard et al. (2017) found research conducted in the Senegalese city of Saint Louis, research participants described the rupture in normal eating and the sensory deprivation of healthy eating as extremely difficult:

“You must stop eating a lot of fat [f. *graisse*]. If you’re old, you should avoid eating just before bed. It important to eat less vegetable oil, to eat less fat. You need to stop eating chilli peppers, stop eating them completely. In fact a lot of things you just have to stop eating completely” [Interview with a woman in her 70s]

When women received diagnoses, most commonly of hypertension or diabetes, they tried to begin to eat ‘healthy’, to lower their consumption of fat, salt and sugar, and to enact the advice they were often given which was to eat less rice. Focus group participants and interviewees identified a ‘traditional’ pattern of eating based on millet and fresh milk that was understood to be much healthier than *plats national*, the dishes shared at midday in Dakar households that acted as conduits for flavour enhancers, increasingly critiqued in Dakar as ‘toxic’. Women reported a range of dietary changes that they used to ‘lower’ [Wolof, *waññi*] their *tension* and to ‘calm’ their diabetes. The majority of research participants with chronic diagnoses had acquired these diagnoses through a single consultation with a doctor following a health crisis. Few people had the resources to afford regular consultation with doctors and in the absence of consistent monitoring, people engaged their own strategies according to their subjective perceptions of their bodily health.

“When my *tension* increased [*yungu*] I stopped my work. I tried to rest [*noppaleku*]. My doctor told me “do this exactly, rest, eat carefully”. I completely stopped eating the foods that were forbidden like sugar. I paid close attention to my blood pressure. But I am still diabetic”. [Focus group participant, 64]

In the focus groups, younger women expressed empathy with people who were managing these symptoms and who experienced pain and social isolation when they had to withdraw from collective eating. However, they also argued that they had to fulfil their social role of nourishing an entire household, from babies to the elderly, and they were obligated to the collective to provide the ‘unhealthy’ food that was stimulating and tasty. Younger participants in focus groups often interpreted the suffering of older people in their households fatalistically, reflecting that they too would eventually and inevitably fall ill because they shared the same diet, the same ‘love’ of bad eating, and the same challenges and standard of living. In the focus groups older people tried to challenge this argument, and to recruit collectives to patterns of healthy eating in which they could be included, by referring to the wisdom and understanding of older people:

“Older people may be more likely to fall ill but they are also the ones that can see furthest [*jeeral*]. Before you decide what is good to eat for yourself, think about your family. What is good for everyone to eat is what is good for health.” [Interview, woman, 72]

Ethnographic data as well as individual interviews with older people revealed that these attempts to persuade households to switch to a different, collective dietary register were rarely successful. In Senegalese households, the main meal is served in the afternoon. In Dakar, this meal is almost always a meal based on rice, either a *benn cinn* (one pot) meal of *cebujen*, a slow cooked paella-style dish of rice and fish, or a *ñaari cin* (two pot) meal of boiled rice with onion or peanut sauce, served with sardines or chicken. Meals are prepared by younger women in the household and shared with everyone present. For the women who prepare food, cooking is much more than a mundane task. It is through cooking that they demonstrate and embody their compliance with the values of Senegalese society. The respect that they are expected to show to their families is materially demonstrated through the preparation of familiar, good tasting and attractive food, and exchanges of food, from the ceremonial and formalised to the everyday, exists within a web of exchanges that dramatizes and materialises relative status and mutual obligation (Yount-André, 2016). For people who wanted to change their diet, the issue of collective eating needed to be negotiated with flexibility and pragmatism. In focus groups and in interviews women reported that they had been told to continue to eat from the rice bowl, but to reduce the amount they had been accustomed to eating. In many cases, the advice to restrict was the primary instruction that women retained from their interactions with doctors. Restriction was intuitive to many of the participants because it already formed part of the complex of control that women had reached through experiments with food. Research participants had reached the conclusion that eating shared food made them experience a spike in negative symptoms, including dizziness, nausea, and thirst. Many women reported that their ‘tolerance’ of their relatives cooking had noticeably declined as they aged and they ate enough to satisfy politeness and to demonstrate respect, but did not eat until they were satisfied [*lekk ba sur*].

Beyond the pragmatic use of restriction to maintain connections to collective eating without compromising individual health, women also knew that they should increase their consumption of fresh fruit and vegetables. As with the advice to take regular exercise, women often pragmatically ‘bracketed’ this advice, acknowledging its relevance and urgency but generally considering that it was not appropriate for the context they dealt with day to day. Fruits and vegetables were relatively expensive and the vegetables that were included in the household meals were often described as being incorporated to enliven or decorate the plate. In addition to ‘banning’ [*tere*], and ‘bracketing’, strategies that were based on the subtraction, substitution, or addition of certain key elements and foodstuffs, women participants in the focus groups had to craft a positive and possible philosophy of healthy eating, largely unaided by medical and dietary advice. In order to do this, they drew on their individual experiences, shared knowledge, and culturally specific understandings of the aesthetic and sensory properties of food that might be ‘good to eat’. Rather than breaking down food into its negative components and enumerating the effects of those items on the body, women spoke about healthy and ideal food conforming to broad local aesthetic categories of ‘plain’ [lëwët], ‘light’ [ouyof], or ‘wet’ [tooy]. However, this poetic and positive translation of uncompromising dietary advice into local ways of assessing food’s efficacy and healthiness did not resolve the high levels of concern that people felt about the safety and healthiness of the food that was available to them. As consumers, as older people, and as members of a vulnerable eating collective, women often summarised their predicament as *amul pexe –* the absence of choice.

## Discussion

Dietary advice has to be interpreted, translated, and enacted within collectives. Each of these steps involves not just individual reflection and the deployment of individual sensory capacities like taste, but also a broader negotiation. In the section above, I have laid out some of the interpretative resources that women bring to bear on dietary advice, how they translate it into a culturally meaningful set of representations, and how they try to make their projects of control, their chronic ‘homework’, a priority for the wider collective. This concern with collective wellbeing and consensus illuminates how chronic ‘homework’ takes on culturally specific dimensions in Senegal, where aspirations are articulated not towards a ‘moral project’ of ‘successful’ aging (Lamb, 2019), in terms of managing the metrics and measurements of bodily performance, but towards the collective care of the body and an aspiration to fulfil a social role of elder person. When embodied experiences such as pain interfere with the fulfilment of these obligations and block exchanges of food and care, relations are disrupted. Disruption in these relationships, in turn, shapes the possibility of controlling and managing chronic disease through attention to the body and strategies of self-care, as this process in Senegalese households is closely linked to personal power: the exercise of efficacy in intimate relationships, the capacity to pursue a medical diagnosis and get the maximum amount of information from healthcare providers, the economic power to access high-quality healthcare at regular intervals and the interpersonal influence and authority to convince or compel other members of one’s household that an unfamiliar and unattractive diet was good to eat and good enough to share. Many research participants living with chronic disease did not succeed in persuading their families that the nutritional value of healthy diets compensated for the loss of culturally meaningful ways of eating. The consequence of this failure was considerable social suffering, as the progress of chronic disease appeared to reflect and reinforce the individual’s disempowerment and diminished social status.

Many older women argued that day-to-day conflict over ‘safe’ nourishment for chronic disease revealed that the Senegalese claim to value the health of older people extremely highly was, in practice, untrue. Confronted by their ‘failure’ to advocate for changed eating and to draw on household resources to source food that they judged was safe for them to eat, the only remaining solution was to limit consumption of shared food, eating less of the rice, sauce, and bread that was served to them at mealtimes. Restriction had social dimensions and impacted on the collective; for young women, it triggered concern that they would be held responsible for older women’s weight loss and physical decline. In one household where I conducted participant observation, one young woman, Mariama,^1^ was extremely concerned by her mother-in-law’s reluctance to eat shared food. Her mother-in-law’s refusal of food constrained Mariama’s own capacity to demonstrate values of respect and modesty and Mariama watched her closely for signs of fatigue, illness, or for changes in the woman’s outward appearance that would signal a decline in her health. Bound to continue cooking according to the preferences of the majority of people in the household, Mariama eventually mobilised her own resources and used her own money to buy food that she found appropriate. Conscious of her mother-in-law’s desires to eat ‘privately’ and wanting to gift something highly valued, pleasurable and satiating to demonstrate her respect, Mariama tended to buy processed foods such as cans of condensed milk. These food items satisfied social and relational needs, but they did not meet nutritional standards of safety and suitability.

The decision to place social and relational needs over nutritional needs can also be strategic. Women diagnosed with chronic disease were aware that their desire to eat different food might be negatively perceived. In this context people used strategies of self-management that were not discussed with medical professionals and that deviated from scripts of healthy eating. Few women asked doctors the questions that really preoccupied them, often because these were questions that would mark them as ‘bad’ patients, willing to bend the rules. Instead, they turned to their peers for practical knowledge sharing about delicate questions to do with the trade-off between nutritional, financial, and emotional resources. As many of the research participants pointed out to me, good health was related to two interrelated but distinct factors: having enough food to eat well day to day and having access to money to mitigate the severity of a health crisis (this distinction was made more complicated by the fact that food was mainly controlled by women [*affairu jigeen*] and money primarily controlled by men). One older woman told me that she accrued more capital in her households through demonstrating her modesty [*kersa*], eating ‘respectfully’ and cooperating with collective consumption. She could then hope that the goodwill generated with her relatives would assure that she would be cared for in a crisis, for example, if she had a stroke or a heart attack. Covert tactics for management like food restriction, humility, and respectful cooperation were often shared in the focus groups. While these could not be directly discussed with doctors because these strategies marked the women who deployed them as non-effective practitioners of self-care, within their households, discretion, evasion, and cooperation were crucial to the management of individual and social diagnoses. Paying attention to ‘mundane social dynamics’ of day-to-day eating in Senegalese households shows how diets are assembled through ‘divisions of labour and negotiations’ (Halkier, 2020), rather than the exercise of individual choice. What could be seen as ‘passive’ strategies of cooperation, modesty and restriction were also framed by women as active ways of practicing solidarity, compassion, and empathy. By not imposing their dietary advice on the collective, they invested in the health of that collective in a broader sense, in its stability, solidarity and cultural coherence, and demonstrated that they should be financially supported by that collective if their health broke down.

## Conclusion

In this paper, I have focused closely on choice, not as a logic embedded in marketised healthcare, but as a daily set of practices and negotiations in a context where individual agency is articulated and experienced through efforts to create and maintain healthy relations. The choices of older women attempting to manage symptoms of chronic disease were guided by an aspiration to fulfil a social role of elder person. When the pain and restriction associated with diabetes and hypertension interfered with the fulfilment of these obligations and blocked exchanges of food and care, people were faced with dilemmas over which forms of eating to prioritise: biomedical and nutritional or social and relational. The close attention paid here to intra-household bargaining and relational personhood in the context of chronicity might seem at odds with the current shift towards ‘upstream’ determinants. However, all upstream determinants are social relations somewhere, and the relationships and behaviours analysed in this paper are all shaped by scarcity, persistent poverty, and fluctuating access to food (Whyte, 2012).

Were a more relational public health to emerge from critical discussion of the challenge of chronicity, it would be less invested in the spatial fixing devices of ‘up’ and ‘down’ stream, instead thinking relationally about how health is constrained and enabled by the dynamic and relational interplay across and between scales. This paper has demonstrated that mobilising individual choice is not a concrete practice that resonates with low-income West African consumers who must consider their own domestic consumption in relation to the needs of the collective. In this context, public health strategies based on changing the food choices that individuals make are highly unlikely to succeed. However, on the basis of the data collected a relational health promotion can certainly be envisaged. This health promotion would be rooted in two parallel and mutually supportive strategies that increase the latitude of social space in which people can exercise and experience agency around food. While a range of interventions promoting dietary diversity, community cooking demonstrations, and the cultivation of public gardens are to be envisaged under this rubric, they should be accompanied by a commitment by governments to social protection that materially supports improvements in nutrition. In so doing, relational public health goes beyond cultural interpretation and attention to nuance and local complexity, and opens up transformative ways of seeing, planning, and evaluating health interventions targeting chronic disease.

## Note

1. All names used are pseudonyms.
